# Protein Corona as a Result of Interaction of Protein Molecules with Inorganic Nanoparticles

**DOI:** 10.3390/ijms26199771

**Published:** 2025-10-07

**Authors:** Ruslan M. Sarimov, Elena A. Molkova, Alexander V. Simakin, Alexey S. Dorokhov, Sergey V. Gudkov

**Affiliations:** 1 Prokhorov General Physics Institute of the Russian Academy of Sciences, 119991 Moscow, Russia; rusa@kapella.gpi.ru (R.M.S.); bronkos627@gmail.com (E.A.M.); avsimakin@gmail.com (A.V.S.); 2 Federal Scientific Agroengineering Center VIM, 109428 Moscow, Russia; dorokhov.vim@yandex.ru; 3 Bauman Moscow State Technical University, 105005 Moscow, Russia

**Keywords:** protein–nanoparticle interactions, protein corona, inorganic nanoparticles, secondary structure, aggregation

## Abstract

Currently, there is a growing interest in biomedical research in the use of inorganic nanoparticles for targeted drug delivery, as biosensors, and in theranostic applications. This review examines the interaction of inorganic nanoparticles with protein molecules depending on the chemical nature, size, and surface charge of the nanoparticles. The effect of protein and nanoparticle concentration, as well as their incubation time, is analyzed. The work focuses on the influence of parameters such as pH, ionic strength, and temperature on the interaction of nanoparticles with protein molecules. The following dependencies were studied in detail: the thickness of the protein corona as a function of nanoparticle size; the size of nanoparticles after interaction with protein as a function of protein and nanoparticle concentration; the distribution of zeta potentials in colloids of nanoparticles, proteins, and their mixtures. It has been shown that proteins and nanoparticles can influence each other’s physicochemical properties. This can lead to the emergence of new biological properties in the system. Therefore, the adsorption of proteins onto nanoparticle surfaces can induce conformational changes. The probability of changing the protein structure increases when a covalent bond is formed between the nanoparticle and the protein molecule. Studies demonstrate that protein structure remains more stable with spherical nanoparticles than with rod-shaped or other high-curvature nanostructures. The results presented in the review demonstrate the possibility of adapting physiological responses to nanomaterials by changing the chemical composition of the surface of nanoparticles and their size and charge.

## 1. Introduction

A wide range of nanoparticles with tailored properties have been developed [1,2,3], and only a small number of these are used to solve practical problems [4,5,6]. Nanomedicine faces numerous issues in the process of clinical trials [7,8]. One of these problems is the interaction of nanoparticles with body components [9,10,11]. After a nanoparticle is introduced into a biological fluid, it interacts with biomolecules such as proteins, lipids, carbohydrates, and nucleic acids [12]. The interaction occurs through the Coulomb force [13], van der Waals [14], hydrogen bonds [15], hydrophobic effects [16], and dispersion forces [17], causing the formation of a “corona” on the surface of particles [18] or even large aggregates [19]. Analysis of publications shows that proteins play a significant role in the formation of the surface coating on nanoparticles among all biomolecules [20]. The rate of formation and configuration of the protein corona depends on many factors, including size [21], shape [22], charge [23], and chemical properties of the particle surface [24].

Understanding the nanoparticle–protein interactions is challenging, as the current knowledge is largely derived from in vitro studies. At the same time, researchers often do not pay attention to the real conditions that exist in cells or tissues of the body in vivo [25]. For example, the study [26] observed that the protein composition on nanoparticles in vivo differed radically from that in vitro and remained stable over time. However, a careful selection of the experimental conditions can yield similar in vitro and in vivo results [27].

Proteins on the nanoparticle surface can act as ligands for specific binding to cells, tissues, or biological molecules [28]. Moreover, the formation of protein corona in cells is often accompanied by a cytotoxic effect [29]. Selection of conditions in in vitro experiments can neutralize the negative effects [28,30,31]. In [32], it was found that manipulation of the composition of the protein corona can affect the interaction of nanoparticles with macrophage cells in culture. In [33], the role of the interaction of proteins and nanoparticles in the development of approaches to the treatment of neurodegenerative diseases associated with amyloidosis was demonstrated. In another study, the addition of gold nanoparticles bound to the APOA1 (Apolipoprotein A1) to endothelial cells leads to a decrease in the expression level of another protein, ZO-1 (Zonula Occludens-1), which indicates the potential possibility of using such nanoparticles to control cellular transcription or translation [34]. Interaction with nanoparticles can affect the native conformation of the protein and, consequently, its functional properties. The presence of nanoparticles can control both the interaction between protein molecules and their enzymatic activity [35,36]. Nanoparticles can also indirectly induce an immune response following a change in protein conformation as a result of interaction [37]. On the other hand, proteins can change the biophysical properties of nanoparticles [38]. Protein adsorption on the surface of nanoparticles imparts new biological properties to the latter [39].

Developing effective nanoparticle applications requires an integrated approach, based on both the precise characteristics of nanomaterials in biological fluids and detailed data on their interactions with proteins. Only in this way can a high-quality database be created, which is necessary for the clinical use of nanoparticles. The study of the interaction of inorganic nanoparticles with cells is also of great importance for drug development [40]. Due to the crucial role of protein–nanoparticle interactions in determining cellular response, numerous studies have focused on formulating conditions for a stable, biocompatible, and non-toxic protein corona.

## 2. Parameters Affecting the Interaction of Nanoparticles with Proteins

### 2.1. Chemical Composition of Nanoparticles

**Figure 1:**
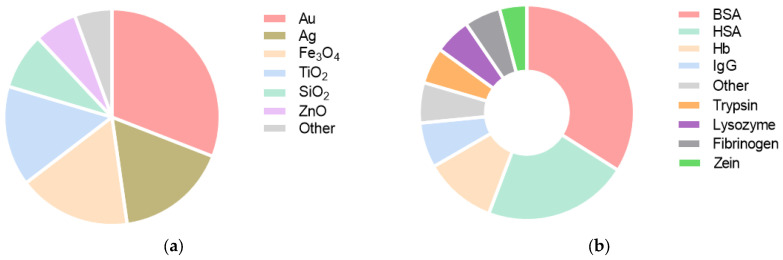
Frequency charts of the use of the main types of inorganic nanoparticles (**a**) and protein molecules (**b**) for studying their interactions in experimental articles over the past 5 years.

An analysis of the frequency of using nanoparticles and proteins in scientific research was conducted (Figure 1). According to the literature, gold and silver nanoparticles are most commonly used in experiments (almost half of the total). Iron and titanium oxide nanoparticles are also frequently used (approximately one-sixth of the total). Silicon oxide and zinc oxide nanoparticles are used less frequently. About 70% of the studies used blood proteins. Among proteins, serum albumin was used in approximately half of the studies. Trypsin, lysozyme, fibrinogen, and zein are also frequently used. Zein is the only plant-based protein that is actively used in studying the interaction of protein molecules with nanoscale objects. Apparently, the use of zein is dictated by its availability and low cost in some countries.

The popularity of using gold and silver nanoparticles is explained by the unique properties of these nanoparticles [41,42,43]. Gold and silver nanoparticles are widely investigated for biomedical applications including biovisualization [44,45], diagnostics and registration of substances and compounds [46,47,48], drug delivery [49], and the fight against microorganisms [50,51]. It is known that the formation of a protein corona in aqueous colloids alters their optical properties. Thus, a change in the position of the plasmon resonance maximum occurs, the efficiency of light absorption and, in some cases, photothermal conversion increases [52,53]. A change in the physicochemical properties can significantly affect the main functionality of the nanoparticles. Thus, the authors associated the appearance of a false positive result in the analysis of a sample for ambroxol hydrochloride with the binding of gold nanoparticles to blood proteins [54]. It should be noted that the formation of a protein corona does not always lead to physicochemical changes in the nanoparticles. For example, in [55] it was shown that gold nanoprisms do not lose their photothermal properties after the formation of a protein corona. Interestingly, the adsorption of human serum albumin (HSA) on gold nanoparticles is largely irreversible [56].

In the work [57], the influence of the size, shape, and surface coating of silver nanoparticles on the interaction with two important transport proteins, HSA and Human α-1-acidglyco-protein (AGP), was assessed. The coating material used for silver nanoparticle stabilization was the primary determinant of silver nanoparticle interactions with plasma transport proteins. The conformational changes in HSA on coated silver nanoparticles were independent of its binding affinity to the nanoparticles. All this indicates the complexity of the process of interaction of proteins with the surface of functionalized nanoparticles. In another case, the formation of protein corona of the AGP protein on silver nanoparticles was accompanied by the preservation of the native conformation of the protein [58]. In [59], it was shown that silver nanoparticles stabilized with polyethyleneimine interact with a high degree of affinity with the BSA (Bovine Serum Albumin) protein and do not interact with IgG (Immunoglobulin G) and lysozyme, which is most likely due to the formation of hydrogen bonds and van der Waals interactions. On the other hand, there is evidence that proteins covering the nanoparticle do not always “shield” the surface of the nanoparticle from the environment. According to [60], BSA can increase the oxidative release of silver ions from nanoparticles in a size-dependent manner. The protein corona of HSA on gold, silver, and platinum nanoparticles changes the antimicrobial activity of the nanoparticles when interacting with E. coli [61]. These results can be used to study and develop innovative approaches to combating the emergence of antibiotic resistance.

**Figure 2:**
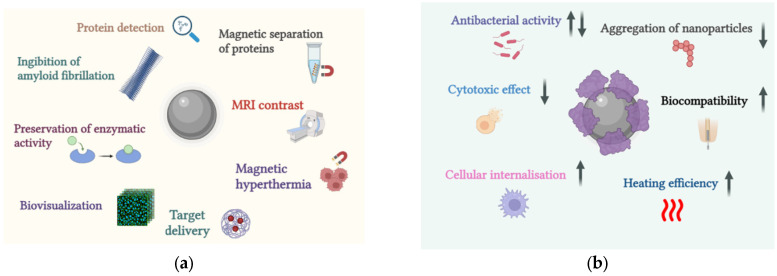
Areas of practical application of nanoparticles in which the interaction of nanoparticles with protein molecules is assumed (**a**). Observed effects when nanoparticles with a protein corona enter the biological environment (**b**). Arrows indicate an increase in the strength of the effect (upward arrow) or a decrease in the strength of the effect (downward arrow).

Magnetic nanoparticles are used in biomedicine mainly for MRI [62], magnetic hyperthermia [63,64], magnetic targeted drug delivery [65], magnetic biodetection [66], and for selective binding and purification of proteins in biological and other samples [67,68]. In general, the areas of application of nanoparticles in which their interaction with proteins is important are shown in Figure 2a. The formation of protein corona can affect the functionality and biocompatibility of such nanoparticles. The main biological effects caused by nanoparticles with protein corona are shown in Figure 2b. It was found that interaction with BSA prevents aggregation and increases the efficiency of heating of iron oxide nanoparticles in a magnetic field [69]. The use of magnetic nanoparticles can increase the accuracy of the process of quantitative assessment of proteins in solutions. In [70], it was proposed to use iron oxide nanoparticles pre-coated with BSA protein to analyze the kinetics of binding to an antibody directly in solution. Immobilization of β-glucosidase on the surface of iron nanoparticles allows maintaining the enzyme’s efficiency during repeated use [71]. This is significant in the food industry for lactose hydrolysis. It is known that some nanoparticles can oxidize surrounding molecules when circulating in biological fluids [72]. This is especially true for nanoparticles of iron oxide magnetites Fe_3_O_4_ and maghemites Fe_2_O_3_, in which reduction processes can occur under the influence of antioxidants: trivalent iron is reduced to divalent. In the presence of peroxides, divalent iron catalyzes the Fenton reaction, generating highly reactive hydroxyl radicals [73]. As a result, oxidative damage to molecules in contact with nanoparticles can be observed. Thus, it has been shown that the adsorption of immunoglobulins on iron oxide nanoparticle surfaces leads to oxidation of their amino acid residues [74]. In contrast, similar iron oxide nanoparticles induce no structural changes in BSA upon interaction [62].

It should be noted that nanoparticles made of noble metals or iron oxides are most often encountered in studies investigating the interaction of nanoparticles with proteins. Obviously, this is due to the prospects for using such nanoparticles for biomedical purposes. However, there are publications studying nanoparticles of silicon, titanium, zinc, aluminum, nickel, copper, and selenium oxides, which also have pronounced biological properties [75,76,77,78,79] (Figure 1). The adsorption of bovine hemoglobin onto silicon nanoparticles revealed high affinity for the hydrophobic surface, accompanied by alterations in its secondary structure, and sometimes heme degradation [80]. In another study, nanoparticles based on mesoporous silicon are used for intracellular delivery of the enzyme superoxide dismutase (SOD) in denatured form [81]. It was shown that after penetration into Neuro-2a cells, the SOD protein restored its specific enzymatic activity.

The most informative and interesting publications compare the interactions of different nanoparticles with one type of protein or different proteins with one type of nanoparticle. For example, when comparing the interaction of the BSA protein with SiO_2_, ZnO, and TiO_2_ nanoparticles, it was shown that the interaction with zinc oxide nanoparticles occurred with the highest degree of affinity [82]. Another study demonstrated that zinc oxide forms more stable conjugates with fibrinogen than with BSA [83]. The chemical nature of nanoparticles significantly influences their effect on metabolic enzymes. Copper oxide nanoparticles have the most negative effect and catalyze the formation of disulfide bonds in catalase, aldolase, LDH and QOR; zinc oxide nanoparticles have a moderate negative effect on enzymes, while aluminum, iron, and nickel oxides have negligible consequences on enzyme structure [84].

It should be noted that different proteins interact otherwise with nanoparticles of different chemical nature, and this can lead to different biological effects. Coating different nanoparticles with proteins is often used to increase the chances of nanoparticle penetration into cells. Thus, the work [85] reported that among the protein coronas formed by zein, BSA, lysozyme, and collagen on titanium dioxide nanoparticles, the lysozyme corona promoted the most efficient absorption of the nanoparticles into SW1417 colorectal cancer cells. Also, when titanium dioxide nanoparticles interact with the proteins β-lactoglobulin and gelatin, the work showed a correlation between the ability of proteins to prevent nanoparticle aggregation and facilitate penetration into cells [86].

Another extensive category of inorganic nanoparticles includes carbon-based materials, specifically fullerenes, carbon nanotubes, graphene, and its derivatives (e.g., graphene oxide) [87]. Due to their unique properties, these materials are being extensively explored for use in diagnostics and biosensor development [88,89]. For example, a carbon nanotube-based biosensor platform has been developed for real-time detection of prostate-specific antigen with a detection limit of 84 pM [90]. In addition, the possibility of preserving enzymatic activity after immobilization is demonstrated, as in the case of galactose oxidase on carbon nanotubes [91]. However, most studies report inhibition of enzymatic activity upon protein interaction with fullerenes [92]. In [93], lysozyme–C_60_ conjugation enhanced the fullerene’s water solubility and resulted in reactive oxygen species (ROS) generation. Molecular modeling reveals that carbon nanoparticles reduce the aggregation propensity of prion proteins by weakening interpeptide interactions and suppressing β-sheet formation [94].

Thus, according to literary sources, over the past 5 years, gold, silver, and iron oxide nanoparticles have mainly been used in experiments. The popularity of using gold and silver nanoparticles is explained by the unique optical properties of these nanoparticles and the abundance of innovative products for diagnostics and detection in biomedical research. Magnetic nanoparticles are used in biomedicine mainly for MRI, magnetic hyperthermia, and delivery. It has been established that the chemical nature of nanoparticles has a significant impact on the processes of formation of the protein corona. It has been shown that both proteins and nanoparticles can affect the physicochemical properties of each other, which can lead to the emergence of new biological properties of the system.

### 2.2. Size of Nanoparticles

Together with the material composition, particle size is a critical factor determining the electronic and optical properties of nanoparticles [95]. This is especially important for monitoring the penetration of nanoparticles into tissues and cells. This is mainly due to the existence of various physiological size thresholds in the body [96]. In general, the rule is that the smaller the particle, the more biological barriers it can overcome. The size of the nanoparticles also determines their reactivity. As the size of the nanoparticle decreases, the following increase: 1. the ratio of the surface area to the volume; 2. the curvature of the surface of the nanoparticles, which often leads to an increase in reactivity [97]. The effect of BSA on the release of silver ions from the surface of nanoparticles with sizes of 10, 20, and 40 nm is considered in [60]. The greatest release of silver ions was observed for nanoparticles with a size of 10 nm. Another study showed that 10 nm silver nanoparticles are more effectively distributed in tissues and cause more serious toxic effects in the liver compared to 40 and 100 nm nanoparticles [98]. The study [99] found that silver nanoparticles with a larger diameter and smaller surface curvature had higher sorption capacity compared to nanoparticles with a smaller diameter. The effect of 5 nm gold nanoparticles on the structure of the pulmonary surfactant protein B analogue was shown to a greater extent compared to larger gold nanoparticles [100]. Apparently, the most effective is the nanoparticle size comparable to the size of the protein molecule. Nanoparticles smaller than the size of the protein molecule are apparently ineffective. Thus, ultra-small gold nanoparticles of ~2 nm have almost no effect on the structure of HSA and transferrin proteins [101]. In cases where the size of the nanoparticles is smaller than the size of the protein molecules, the use of the term “protein corona” is probably inappropriate.

### 2.3. Surface Properties of Nanoparticles

**Figure 3:**
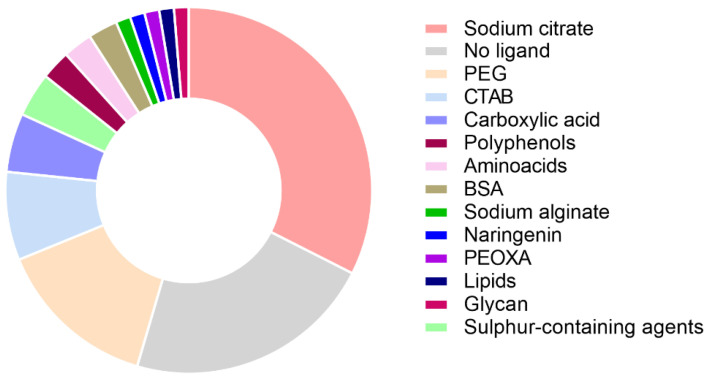
Diagram of the frequency of use of different chemical compounds for coating the surface of nanoparticles in experimental articles over the past 5 years.

The formation of the protein corona is strongly influenced by the physicochemical properties of the nanoparticle surface, which directly interacts with protein molecules [102]. The surface of nanoparticles can be coated or functionalized with various compounds (Figure 3). As a result, one can achieve significant improvements in the bioavailability and stability of the nanoparticle drug [103,104]. Also, the functionalization of nanoparticles helps to reduce the cytotoxicity of nanoparticles [105]. Functionalizing magnetic nanoparticles with an aptamer ensures optimal spatial orientation of the immobilized LECT2 (Leukocyte cell-derived chemotaxin-2), enabling an SPR analysis sensitivity of up to 10 pg/mL [106]. Nanoparticles with identical material, size, and shape can exhibit distinct protein interactions due to variations in their surface chemical groups. A significant loss of the α-helix structure by hemoglobin was observed upon interaction of the protein with sodium citrate-coated gold nanoparticles [107]. However, minimal changes in the secondary structure of the protein were observed upon interaction of Hb (hemoglobin) with the same gold nanoparticles with a sulfonate-modified lipoic acid ligand. In [108], the authors observed the effect of silicon nanoparticle functionalization on the production of proinflammatory cytokines. Nanoparticles functionalized with 1,7-octadiene caused an increase in the synthesis of proinflammatory cytokines by macrophages in various amounts, namely IL-6 (1200 pg/mL), IL-1b (1050 pg/mL), and TNF-α (5900 pg/mL). Nanoparticles functionalized with a carboxylic acid residue increased the production of IL-6 cytokines (1200 pg/mL). On the other hand, functionalization with allylamine increased the expression of arginase (5800 pg/mL), IL-1RA (5900 pg/mL), and IL-10 (32 pg/mL).

The charge or hydrophobicity/hydrophilicity state on the surface of nanoparticles plays a major role during interaction with many proteins [109]. Zwitterionic functionalization enables silicon nanoparticles to bind proteins without inducing structural changes; in practice, this allows them to overcome the barriers of the gastrointestinal tract [110]. Notable exceptions to this trend include apolipoproteins, which exhibit the ability to interact with various nanosurfaces regardless of surface hydrophobicity/hydrophilicity [111].

Although a higher surface charge modulus generally enhances nanoparticle stability [112], it does not always improve their performance in biomedical applications. For example, it is known that cationic nanoparticles can induce immunological reactions in cells, which limits their application [113]. Another strategy in the development of functionalized nanoparticles is to “mask” the nanoparticles from non-specific protein adsorption by modifying the particle surface with hydrophilic polymers, especially PEG (polyethylene glycol) [114]. The development of nanoparticles that can completely avoid protein binding can improve the performance of synthetic nanomaterials in physiological environments and create a variety of potential applications in materials science and biotechnology [115].

Functionalization of nanoparticles with PEG can alter the surface charge and sterically hinder protein adsorption. However, PEG has been shown to minimize protein adsorption but does not completely suppress it, and repeated exposure to PEG in vivo induces the production of PEG-specific antibodies [116]. The composition and thickness of the protein corona formed from serum on oligoethylene glycol-functionalized silver nanoparticles were found to be governed by the surface density of the oligoethylene glycol ligands [117]. The amount of adsorbed protein decreased with an increase in the density of oligoethylene glycol molecules on the surface of the nanoparticles. Nevertheless, complete surface coverage with oligoethylene glycol was insufficient to fully suppress protein adsorption. In [118], the authors show that the formation of protein corona from HSA can be quantitatively suppressed by functionalizing iron oxide nanoparticles with cyclic poly2-ethyl-2-oxazoline. Dense shells of linear poly2-ethyl-2-oxazoline molecules cannot prevent weak interactions with HSA. It was found that silver nanoparticles functionalized with poly(2-vinyl pyridine)-b-poly(ethylene oxide) and polyvinylpyrrolidone are resistant to the adsorption of IgG proteins and lysozyme, but not BSA [59]. In [119], gold nanoparticles are effectively functionalized with glutathione monoethyl ether to increase the ability to resist protein adsorption and aggregation stability. The formation of a lipid coating on gold nanoparticles functionalized with phenylalanine provides resistance to the adsorption of proteins with different charges [120]. The authors of [121] showed that functionalization of the surface of nanoparticles with the amino acids aspartic acid and serine prevents trypsin adsorption on TiO_2_ nanoparticles.

It is important to note that surface PEGylation often leads to a stealth effect that helps nanoparticles avoid recognition and rapid clearance by the RES (reticuloendothelial system) [122]. The implementation of stealth approach plays a crucial role in nanomaterial-based drug delivery by enhancing pharmacokinetic properties such as prolonged circulation, optimized biodistribution, and tissue-selective accumulation [123]. As discussed above, PEGylation does not always completely suppress protein adsorption. The effectiveness of this strategy may directly depend on the interaction of nanoparticles with blood proteins. The rate of nanomaterial removal from the blood is significantly affected by changes in the protein structure when interacting with a nanoparticle [124]. Control over the formation of the protein crown is important for the successful application of stealth effect.

Osmolytes are used to prevent protein aggregation by altering the properties of biological fluids, though they require high concentrations to be effective (more than 1 M). Contemporary studies indicate a synergistic effect where nanoparticles significantly potentiate the ability of osmolytes to suppress protein aggregation [125,126]. There are publications on the functionalization of the surface of nanoparticles with osmolytes to improve their biocompatibility [127]. Functionalizing iron oxide nanoparticles with trehalose enhanced the structural stability of lysozyme in biological environments [128]. At the same time, the authors obtained the opposite effect in a study using proline to functionalize zinc oxide nanoparticles [129]. When conjugated with zinc oxide nanoparticles, proline loses its potential as an antiaggregating osmolyte and, on the contrary, contributes to an increase in the number of large aggregates during thermal denaturation of BSA. It is also possible to functionalize the surface of a nanoparticle with peptide ligands. In [130], the authors demonstrated the possibility of creating an artificial antibody by grafting complementary-determining regions onto the surface of a gold nanoparticle, which can bind to various sites in lysozyme. Pre-functionalization of upconversion nanoparticles with denatured BSA protein reduces the undesirable effects of protein corona formation when entering biological environments [131].

The results obtained indicate the possibility of adapting physiological reactions to nanomaterials by engineering the chemical composition of their surface in accordance with a specific application.

### 2.4. Physicochemical Properties of the Colloid

**Figure 4:**
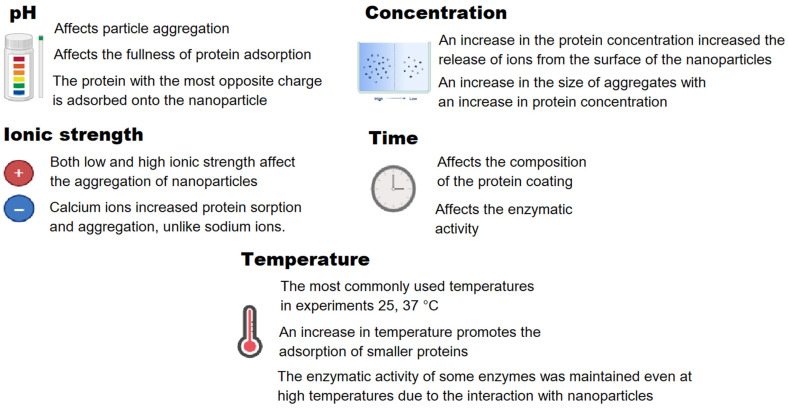
The main physical and chemical properties of the colloid allow control of the interaction of nanoparticles with protein molecules.

A large number of works are devoted to attempts to control the adsorption of proteins on the surface of nanoparticles by influencing the physicochemical properties of the colloid. Figure 4 shows the main factors by changing which it is possible to influence the degree of interaction of proteins with nanoparticles in colloidal solutions.

It is known that the interaction scenario of protein molecules with nanoparticles depends to a significant extent on the pH of the colloid in which the proteins and nanoparticles are located. The pH indicator is the negative logarithm of the proton concentration in the medium, it is a measure of the proton concentration. An increase or decrease in the proton concentration changes the properties of the double electric layer and directly affects the electrokinetic potential of the particles in the colloid [132]. The electrokinetic potential is a measure of the stability of the colloidal system, which affects the probability of flocculation or coagulation [112]. The electrostatic repulsion between proteins is relatively weak near the isoelectric point (IEP), and the adsorption of proteins on the surface of nanoparticles can be most intense [133]. The authors define the adsorption of BSA on iron oxide nanoparticles as pH-dependent and maximum at pI of the BSA protein (0.39 g per 1 g of magnetic nanoparticles) [134]. In contrast, protein adsorption decreases at pH values far from the isoelectric point due to strong electrostatic repulsion between adsorbed proteins and those approaching the surface [135]. In [136], negatively charged TiO_2_ nanoparticles interact more strongly with lysozyme (pI ~ 11.0) at low pH values compared to BSA (pI ~ 4.9) [137]. Solution pH significantly influenced the aggregation behavior of gold nanoparticles during their interaction with IgG (pI ~ 6.0–8.0); at pH < 7.5, particle aggregation occurred [30]. Both increasing pH from slightly acidic (~6.0) to slightly alkaline (~7.5) conditions and elevated ionic strength reduce the stability of the HSA (pI ~ 4.7) protein corona on magnetic nanoparticles [138]. The authors of [139] emphasize that pH changes have minimal effect on serum proteins already adsorbed onto chromium oxide nanoparticles. In work [140], a mechanism for controlling the protein adsorption of myoglobin (pI ~ 7.0) on silica nanoparticles by changing the pH together with the ionic strength of the medium is described. Electrostatic screening by NaCl at pH < pI reduces repulsive forces between surface-adsorbed and solution-phase proteins, enabling the formation of multilayer protein corona. A myoglobin monolayer is observed on the surface of silica nanoparticles with increasing pH > pI upon the addition of NaCl.

In addition to pH, the ionic strength of the solution has a great influence on the interaction of nanoparticles and proteins. The affinity of interaction of nanoparticles with protein can be adjusted by changing the composition and concentration of the buffer in the solution [141]. This effect is highly dependent on the specific system. Low ionic strength selectively enhances the adsorption of specific IgG segments and promotes silver nanoparticle aggregation in systems containing fibrinogen and IgG [142]. Silver nanoparticle aggregation is jointly suppressed by IgG and fibrinogen under high ionic strength conditions. In contrast, high ionic strength during BSA adsorption on SiO_2_ induces nanoparticle aggregation [19]. This is likely due to the shielding of electrostatic interactions, which reduces surface charge and weakens interparticle repulsion. The nature of ions also influences the interaction of proteins with nanoparticles. Calcium ions, unlike sodium ions, facilitated additional HSA adsorption onto gold nanoparticles by formation of -COO-Ca^2+^ bonds, and ultimately enhanced the aggregation between nanoparticles [56].

Ambient temperature is one of the significant factors in the interaction of nanoparticles and proteins. Temperature affects the degree of protein coverage and the composition of adsorbed proteins on the surface of nanoparticles [143]. The researchers often choose a temperature of 37 °C if the potential use of nanoparticles is planned in in vivo experiments [144]. In vitro research frequently uses room temperature [43,145,146]. In [101], the authors show different effects of temperature on the interaction of gold nanoparticles with HSA and transferrin. The binding constant with HSA increased from 3.8 to 8.6 × 10^5^ M^−1^ with an increase in temperature from 21 °C to 35 °C, and with transferrin it decreased from 5.3 to 4.2 × 10^5^ M^−1^. The authors [147] demonstrated that BSA adsorbed on magnetic nanoparticles at 30 °C undergoes more extensive desorption (>80% efficiency) than when adsorbed at 40 °C. Conjugating papain to carboxymethyl chitosan-coated iron oxide nanoparticles prevents thermal inactivation, preserving up to 95% enzymatic activity after 7 h at 55 °C, compared to only 25% for the native enzyme [148]. The enzymatic activity of alcalase 2.4L protease immobilized on chitosan-coated magnetic nanoparticles remained significantly higher than that of the native enzyme within the 55–70 °C temperature range [149]. In similar work, the authors show that the activity of lipase immobilized on chitosan-coated iron nanoparticles is also 10–40% higher at temperatures of 55–80 °C compared to native lipase [150]. The authors in [151] showed that myoglobin adsorption on magnetic nanoparticles is an endothermic process and increases with rising temperature from 10 to 30 °C. Incubation temperature affects the composition of the protein corona and the degree of protein adsorption on nanoparticles [152]. However, the authors showed minimal effect of heating to 45 °C on the surface charge of protein-coated nanorods [153]. Our study examined how temperature affects gold nanoparticle-lysozyme complex size [154]. Increasing the temperature from 25 °C to 85 °C reduced large aggregates size from 650 nm to 160 nm at pH 7.5. In contrast to gold nanoparticles, iron oxide nanoparticles formed larger aggregates with BSA as temperature increased. At 25 °C the hydrodynamic diameter was 25 nm (pH ~ 5.0), and at 85 °C 1.5 µm [155]. At pH 8.0 and 25 °C the hydrodynamic diameter was 13 nm and with increasing temperature to 85 °C the size increased slightly to 40 nm. The authors of the review [156] distinguish three classes of proteins based on the type of temperature effect on interaction with nanoparticles. Most proteins ~65% do not change their affinity for nanoparticles with temperature changes in the range from 4 to 47 °C, ~17% of proteins change their binding to nanoparticles at temperatures >30 °C, and ~18% of proteins change their binding to nanoparticles at temperatures <30 °C.

Another important factor for the interaction of nanoparticles and proteins is the incubation time. Reference [157] revealed that incubation time differentially affects the enzymatic activity of CAT (catalase) and SOD (superoxide dismutase). The enzymatic activity of SOD does not change when interacting with silver nanoparticles even after 24 h. At the same time, the enzymatic activity of CAT after 3 h of incubation with silver nanoparticles decreased by 17%, and after 24 h by 40%. According to [83], the hydrodynamic diameter of ZnO nanoparticles with fibrinogen decreased from 30 nm after 2 h to 25 nm following 24 h incubation. The same study demonstrated that the size of aggregates BSA with nanoparticles grows as the incubation time extends to 24 h, regardless of the initial size of the nanoparticle. Reference [158] examined how incubation time with BSA-coated silicon nanoparticles affects cell association. THP-1 macrophage association decreased by 50% within 15 min and remained stable over 6 h, while hCMEC/D3 cell association showed a gradual decline of 30% at 15 min and 40% after 6 h. The composition of the protein corona depended on the incubation time after ex vivo administration of AuNPs [159].

The concentrations of both proteins and nanoparticles are also determining factors. An increase in the BSA concentration from 0.5 nM to 2 nM in a colloidal solution of silver nanoparticles led to an increase in the oxidative release of Ag(Ⅰ) ions from the surface of the nanoparticles [60]. An increase in the APOH (Apolipoprotein H) concentration from 1 µg/mL to 150 µg/mL in a colloidal solution of silicon nanoparticles led to the formation of additional APOH protein layers on the nanoparticles [158]. An increase in the concentration of TiO_2_ nanoparticles from 50 µM to 100 µM during interaction with the HEWL (Hen egg white lysozyme) led to an increase in the particle size from 195 nm to 445 nm in the colloid [160]. Reference [161] demonstrated HEWL interactions with iron oxide nanoparticle at concentrations as low as 0.01 mg/mL protein and 10^11^ particles/mL nanoparticles. An increase in the size complex of protein with nanoparticles was observed with an increase in the concentration of nanoparticles to 10^13^ particles/mL and with a decrease in the protein concentration to 0.01 mg/mL. The latter trend results from multiparticle binding to individual protein molecules under protein-deficient conditions, which promotes aggregation. Unlike this system, a greater number of proteins were adsorbed on magnetic nanoparticles with an increase in the concentration of FBS [162]. For 1% serum on nanoparticles, 5 µg of proteins were adsorbed per 1 mg of nanoparticles, and for native serum (100%) already 30 µg of proteins per 1 mg of nanoparticles.

The modulation of physicochemical conditions in colloidal systems provides a means to regulate protein–nanoparticle interactions. Adjusting the pH and ionic strength of the solution allows for controlling the extent of protein adsorption onto nanoparticles and particle aggregation. The most significant interaction with nanoparticles is observed at the isoelectric point of the protein. Although raising temperature to 50 °C has little impact on binding affinity, it substantially modifies protein corona composition and protein–nanoparticle complex size. Enzyme immobilization on nanoparticles improves thermal stability and suppresses temperature-induced structural changes. That is, the protein structure is less susceptible to changes during immobilization on the surface of the nanoparticle as the temperature increases. Conversely, longer incubation times (up to 24 h) typically reduce enzymatic activity while increasing protein adsorption on nanoparticles. Similarly, an increase in protein concentration leads to the formation of additional protein layers on the nanoparticles and an increase in the size.

## 3. Influence of Nanoparticles on Protein Structure

Interaction with nanoparticles frequently alters the native structure of protein macromolecules, potentially leading to loss of biological function [163]. Changes in protein conformation can lead to its aggregation and/or incorrect interactions with other cellular components [164,165]. The resulting loss of cellular viability may progress to complete cell death [166,167]. Incorrect folding of the protein polypeptide chain or aggregation is closely associated with such serious degenerative human diseases as Alzheimer’s disease, amyotrophic lateral sclerosis, etc. [168,169]. Understanding protein refolding and preventing aggregation remain persistent challenges driving scientific investigation.

Fourier transform infrared spectroscopy (FTIR), circular dichroism (CD), and fluorescence spectroscopy are used to determine changes in protein structure [170,171,172,173,174]. The utility of FTIR for protein analysis is constrained by dual obstacles: mandatory high sample concentrations (>~10 mM) and water’s interfering IR absorption [175]. While desiccation addresses the latter, it simultaneously disrupts the protein’s hydration shell altering the protein’s native structure and spectral characteristics [176]. The use of fluorescence spectroscopy is often used as an additional research method to observe hydrophobic/hydrophilic changes around protein fluorophores [177].

Interacting systems typically show mutual influence [178], and the nanoparticle–protein system is no exception. Morphologically distinct silver nanoparticles modify BSA conformation [179,180] and promote hemoglobin structural transitions characterized by increased β-sheet formation and decreased α-helix content [181]. Similar structural changes are observed for gold nanoparticles: upon formation of Au-S bonds with thiol groups of proteins, the proportion of α–helices in BSA decreases [182]. Such bonding may induce conformational changes in protein secondary structure [15]. The interaction between BSA and gold nanoparticles has been experimentally confirmed to reduce the protein’s α-helical content [31]. In another study, significant loss of the α-helix structure by hemoglobin was noted during interaction with gold nanoparticles [107]. It should be noted that conformational changes in proteins during interaction with nanoparticles demonstrate a general tendency to decrease alpha-helicity.

Gold nanoparticle shape modulates the structural integrity of fibrinogen and trypsin [183]. Nanorods and nanostars caused more significant changes in the structure of proteins, compared to spherical nanoparticles. The effect on the structure of trypsin was more pronounced than on the structure of fibrinogen. Rod-shaped gold nanoparticles likewise induced structural reorganization of HSA, characterized by reduced α-helical content and increased β-sheets [184]. The same authors continue a series of studies investigating the effect of shape and temperature on the properties of the protein corona of HSA [185]. It was shown that nanorods exhibit a greater tendency to aggregate when interacting with HSA compared to nanospheres. Temperature-induced conformational changes in HSA secondary structure were significantly more evident with nanorods than with spherical nanoparticles. The effect of rod-shaped gold nanoparticles on the microenvironment near the amino acid residues of tryptophan and tyrosine of proteins was also shown in [186]. Gold nanoflowers demonstrate the smallest changes in protein conformation compared to gold nanorods and nanospheres [187]. The binding of gold nanorods to BHb (bovine hemoglobin) and EMb (equine skeletal myoglobin) causes conformational changes in the structure of these molecules [188]. Bimetallic Au/Ag alloy nanoparticles cause conformational changes in BHb and lysozyme proteins [189]. In contrast, biopolymer-functionalized gold nanoparticles significantly suppressed the structural transition from α-helices to β-sheets, characteristic of the formation of insulin amyloid fibrils [190]. Titanium dioxide nanoparticles have been shown not to affect the secondary structure of pepsin, but affect the activity of the enzyme [35]. This is often observed with nonspecific binding of proteins and nanoparticles and is explained by the steric limitations of the active sites of protein molecules when binding to nanoparticles. Nanoparticles can block enzyme active sites through specific binding geometries that occlude substrate entry. But this does not always observe. Previous experiments demonstrated that iron oxide nanoparticles and lysozyme form large micron-sized aggregates without loss of enzymatic activity [161]. In the works of other authors, aluminum oxide nanoparticles had almost no effect on the structure of HSA at nanoparticle concentrations up to 50 μg/mL [191], and the addition of chromium oxide nanoparticles did not lead to significant changes in the structure of BSA [192]. However, more often nanoparticles significantly modify the protein structure. In work [193], significant changes in the native structure of luciferase were observed upon addition of TiO_2_ nanoparticles to the solution. Nanoparticles also contribute to the incorrect renaturation of initially denatured luciferase. The protein structure changes upon adsorption of β-lactoglobulin onto the surface of titanium dioxide nanoparticles [86]. Silicon dioxide nanoparticles accelerated the formation of amyloid fibrils of α-synuclein [194]. The content of β-structures and disordered conformations increases due to the content of the α-helix upon interaction of BSA with titanium dioxide nanoparticles [195]. The interaction of membrane proteins with titanium dioxide nanoparticles was accompanied by protein denaturation [196].

There are known studies in which nanoparticles, on the contrary, stabilized the structure of proteins. Gold nanoparticles functionalized with proline prevented the formation of amyloid fibrils by stabilizing the native structure of insulin during chemical denaturation [126]. Functionalized with naringenin gold nanoparticles prevented the formation of amyloid fibrils [197]. In work [36], after interaction with gold nanoparticles at alkaline pH values lysozyme molecules exhibit significantly higher catalytic activity compared to the control. Reference [198] demonstrated that iron oxide nanoparticles dose-dependently enhanced lysozyme renaturation following guanidine hydrochloride denaturation. On the other hand, iron oxide nanoparticles suppressed protein renaturation and doubled the denaturation rate in the case of the EGFP protein [199].

Fetal bovine plasma or serum are often used as a model biological medium. The greatest ability to bind to nanoparticles is often observed in BSA, Hb, and figrinogen protein molecules. It has been shown that the interaction of SiO_2_ nanoparticles with BSA and hemoglobin led to loosening and unfolding of the peptide chain, while the secondary structure of fibrinogen remains unchanged upon adsorption of SiO_2_ nanoparticles [200].

It can be argued that the binding of nanoparticles to protein molecules significantly increases the chances of changing the secondary structure of the latter. Spherical particles are less likely to affect the secondary structure of a protein compared to rod-shaped or any other nanoparticles with sharp ends. The same nanoparticle can affect the secondary structure of one protein and not affect the secondary structure of another.

## 4. Binding Constants of Proteins with Inorganic Nanoparticles

Protein binding to nanoparticles can be characterized using various parameters, including kinetic and thermodynamic ones [201,202]. The degree or affinity of protein interaction with the surface of nanoparticles can be assessed using physical parameters, including the binding constant. The binding constant value is often determined using optical methods such as absorption and fluorescence spectroscopy, differential scanning calorimetry [101,203].

The interaction of the protein with the nanoparticles occurs due to the interaction of the surface of the nanoparticles with the amino acids that are part of the protein. High affinity of amino acids that are highly soluble in water (valine, proline, and lysine) for iron oxide nanoparticles has been shown [204]. The poor solubility of tyrosine and glutamic acid in water apparently limits adsorption on nanoparticles. The presence of an external thiol in the protein structure (e.g., a cysteine residue) leads to an increase in the affinity of the protein for the surface of gold nanoparticles through the formation of a covalent bond. Reference [182] reports a high affinity for the surface of HSA proteins due to the free thiol. Work [102] shows that the binding constant of BSA and fibrinogen to gold nanorods depends on the coating that stabilizes the particles. These whey proteins bound to cetyltrimethylammonium bromide (CTAB) coated nanoparticles with high affinity (~ 10^8^–10^9^ M^−1^). The degree of protein binding decreased when coated with PEG, ~10^4^–10^6^ M^−1^, but the protein corona was still formed.

The binding affinity of HSA to iron oxide nanoparticles also depends on the functionalization of the nanoparticles. Moderate binding constants of ~10^4^ M^−1^ were observed for uncoated nanoparticles [138], in contrast to ~10^8^ M^−1^ for functionalized nanoparticles [62]. The commonly encountered functionalization of iron oxide nanoparticles with sodium citrate shows moderate binding constants of ~10^5^ M^−1^ for the interaction of nanoparticles with the structurally similar protein BSA [205].

The effect of protein size on binding affinity to silicon nanoparticles was shown in [206]. High binding constants were observed in cases with large proteins. Low binding constant values of ~10^2^–10^4^ M^−1^ of BSA with silicon, titanium, and zinc oxide nanoparticles are reported in publications [82,129,136,207]. Many studies report high binding constant values of ~10^6^–10^9^ M^−1^ [55,59,60,180,208] for the interaction of BSA in micromolar concentrations with silver and gold nanoparticles. A study of the interaction of biosynthesized silver nanoparticles with HSA showed a moderate binding constant of ~10^4^ M^−1^ [209]. High binding affinity of ~10^7^ M^−1^ is shown by zein and casein with zinc oxide nanoparticles [210].

The values of the binding constants of nanoparticles and proteins vary in a wide range of 10^4^–10^9^ M^−1^. The binding constant increases in cases of interaction of nanoparticles with large proteins, and in cases of interaction of proteins with gold and silver nanoparticles. The binding constant strongly depends on the functionalization of the nanoparticle surface. It should be emphasized that each case requires careful consideration and analysis.

## 5. Sizes of Complex of Nanoparticles with Proteins and the Thickness of the Protein Corona on the Surface of Nanoparticles

There are many methods for studying the size of nanoparticles: atomic force microscopy, scanning ion occlusion sensing, nanoparticle tracking analysis, differential centrifugal sedimentation, and scanning mobility particle sizing. However, we will focus in more detail on the advantages and disadvantages of TEM and DLS, as these two methods are most commonly used in work with nanoparticles and proteins. Particle sizes obtained by DLS and TEM often differ from each other [121,211,212].

Among these two methods, authors often prefer TEM microscopy. A leading publication on nanoparticles is not complete without transmission electron microscopy (TEM) photographs. However, the use of TEM is not without limitations and pitfalls. Aside from the cost of the equipment, the biggest limitation of TEM is that it only allows for viewing the sample of nanoparticles in the dry state on the substrate [213]. During drying, the nanoparticles may agglomerate or the initial clusters may break down. Another problem is the limited data presented in a TEM photograph, where only a small part of the sample is visible (<0.1%). The statistical representation of large particles in TEM characterization is constrained by the small sample size compared to the entire ensemble [214]. However, the undoubted advantage of the TEM method is the ability to detect the formation of a protein monolayer on nanoparticles even during the formation of large aggregates [134,161].

The DLS method also has its own advantages and disadvantages. Its benefits include relatively low cost and speed. A clear advantage of the DLS method is the determination of particle sizes: 1. directly in colloidal solution without drying the sample; 2. on average over the entire sample. DLS shows greater variability than other methods, even for silica particles [215]. In addition, DLS poorly determines particle size in solutions with a high polydispersity index (PDI > 0.2–0.3) [216,217]. A main problem that many researchers overlook is that DLS measures the hydrodynamic diameter of molecules, not their actual size. The fact is that the hydrodynamic diameter (radius) is a calculated value that reflects the diffusion of spherical particles in a liquid, and diffusion depends heavily not only on the size and geometry of the particle, but also on the parameters of the medium. X-ray analysis may not always give the same size as the hydrodynamic diameter of particles. This means that the output data obtained in DLS will depend on the interaction between the particles and the solvent. For example, the hydrodynamic diameter of the lysozyme protein in water is smaller than in salt buffers [218] and than the size obtained by X-ray structural analysis [219]. This feature of the DLS method was noted earlier [220] and also observed by us earlier [221]. When applied to the interaction between nanoparticles and proteins, and to the formation of a corona, the hydrodynamic diameter of the resulting corona around a nanoparticle depends not only on the thickness of the protein corona, but also on the stiffness of the corona itself [220]. While DLS is quite sensitive to large particles [222], it does not always provide information about the formation of a monolayer on the surface of nanoparticles when studying the interaction of nanoparticles with proteins.

Considering the advantages and disadvantages mentioned earlier, these two frequency methods are combined to obtain more complete, reliable, and reproducible data.

**Figure 5:**
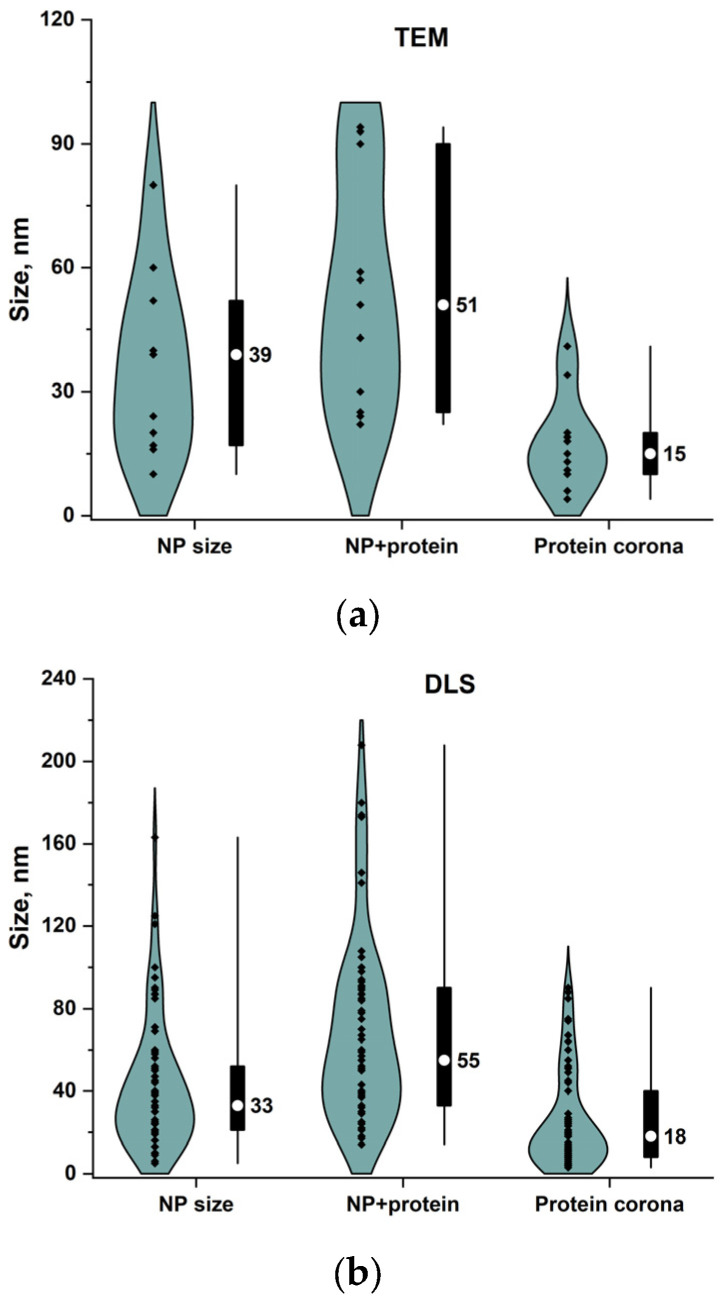

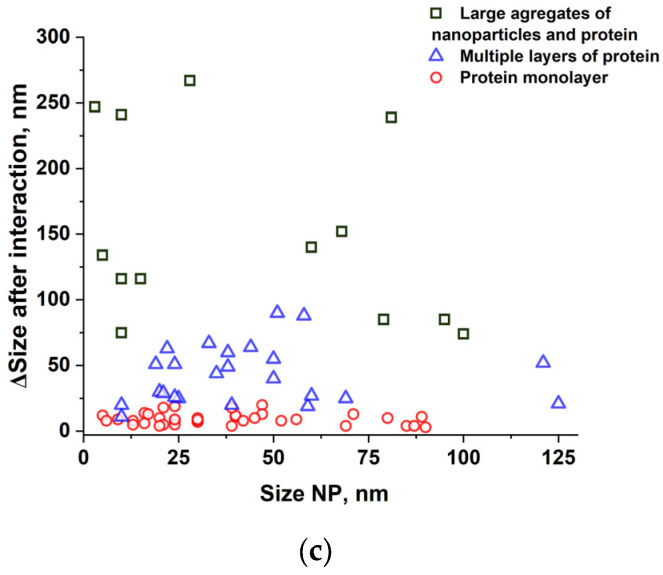
Protein corona thickness as a function of nanoparticle size. (**a**) TEM analysis of nanoparticle size changes after interaction with protein. (**b**) DLS analysis of nanoparticle hydrodynamic diameter changes after interaction with protein. * Not only a protein corona can be formed, but also large aggregates of protein and nanoparticles are found in the case of DLS measurements. (**c**) Increase in nanoparticle size after interaction with protein molecules (abscissa shows nanoparticle size before interaction with protein, while ordinate shows an increase in size after interaction). The values of protein monolayer/multiple layers and aggregate sizes were obtained based on the difference in hydrodynamic diameters of protein-containing nanoparticles and protein-free nanoparticles (Table 1). The white circle in the box is the median; the box is 25–75%; the whiskers are the range.

From the data in Table 1, compiled from more than 80 articles, we calculated the average value of the thickness of the protein corona on nanoparticles or the size of the complex of nanoparticles with protein. Figure 5a,b show the sizes of the following: 1. nanoparticles used in the experimental articles; 2. protein–nanoparticle complex; and 3. protein corona on nanoparticle or the size of the complex of nanoparticles with protein. To distinguish the protein corona thickness (Figure 5a) from nanoparticle–protein aggregates (Figure 5b) in colloidal solutions, we present comparative data from TEM and DLS measurements. The median size of the nanoparticles used in the experiments is about 33–39 nm. At the same time, the median size of the nanoparticle–protein complex is about 51–55 nm. Thus, the size of the protein–nanoparticle complex increases by an average of ~50% compared to the size of the nanoparticle. Simple arithmetic calculations yield an estimated protein corona thickness of 15–18 nm. Figure 5c shows that 1. mainly large aggregates of nanoparticles with proteins form small nanoparticles (up to 30 nm, gray dots), 2. protein monolayer is not formed on nanoparticles larger than 100 nm, 3. protein monolayer on nanoparticles in 80% of cases is formed on nanoparticles up to 50 nm in size, 4. in 60% of cases, the thickness of the protein corona is no more than 30% of the nanoparticle size, 5. while in 80% of cases the protein corona on the nanoparticle is no more than 100% of the nanoparticle size, this is approximately 4–40 nm on particles 5–50 nm in size.

**Figure 6:**
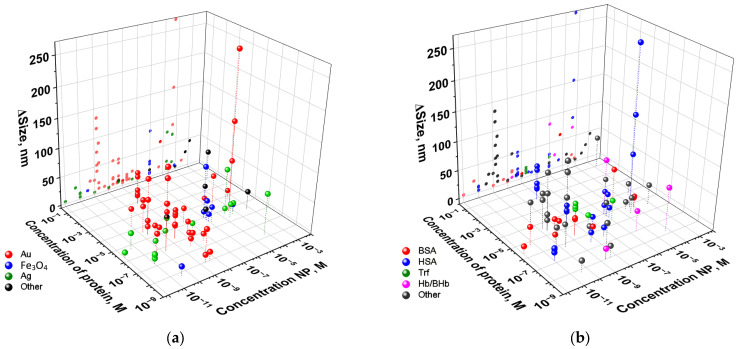
Change in the size of nanoparticles after interaction with protein, depending on the concentration of proteins and nanoparticles in the colloid. Analysis of data by chemical composition of nanoparticles (**a**) or protein type (**b**).

Figure 6 shows the dependence of the size of the protein corona formed on the nanoparticle or their aggregates on the concentration of proteins and nanoparticles in the colloidal solution. Most investigations of nanoparticle–protein interactions employ concentrations between 10^−10^ to 10^−5^ M. It is worth noting that nanoparticles are not molecular substances, and expressing their concentration in mol/L units is not correct. However, the vast majority of articles provide the concentration of nanoparticles in molar concentration, and the authors do not explain how they calculated this concentration. For example, the authors of the article [242] emphasize that the following assumption can be made for gold nanoparticles: the number of moles is equal to the number of gold atoms divided by the Avogadro constant, and not the number of nanoparticles divided by the Avogadro constant. Protein concentrations are often 1–3 orders of magnitude higher than the concentration of nanoparticles (Figure 6a). Authors often describe the necessity of exceeding the protein concentration for maximum adsorption and saturation of the nanoparticle surface with protein molecules. In bloodstream conditions, proteins maintain a significant concentration advantage over administered nanoparticles, with molar ratios frequently exceeding more than 3 orders. In this regard, such a protein/nanoparticle ratio can be considered adequate. As shown in Figure 6a, gold nanoparticles are commonly used at 10^−10^–10^−7^ M concentrations. So, the protein concentration can be much higher, and the maximum size of the nanoparticle aggregate with protein is observed only with a significant excess of protein concentration on nanoparticles. In a large number of cases, an increase in the nanoparticle size by more than 40 nm occurs with the HSA protein, and at fairly low concentrations of ~10^−6^ M (Figure 6b).

## 6. Change in the ξ-Potential of the System During the Interaction of Proteins with Nanoparticles

The protein corona formed on nanoparticles can significantly change the properties of the nanoparticle surface, including the charge. Therefore, researchers often measure the zeta potential and use it as a parameter to describe the stability of the colloidal system [243]. Theoretical modeling [244] revealed that the maximum energy barrier for iron oxide nanoparticle interactions occurs at pH values distinct from those yielding the highest ζ-potential and minimal particle size. This demonstrates that elevated ζ-potential values do not necessarily correlate with enhanced colloidal stability.

Table 1 presents the experimental data measured for the colloid of nanoparticles and the colloid of nanoparticles interacting with protein. The charge of protein has rarely been determined experimentally; in most of the analyzed papers, the determination of this value was often neglected. The zeta potential value at a given pH of the colloidal solution was obtained from the literature for some proteins [245,246]. But for the remaining proteins, the zeta potential value was determined by extrapolation from the pH and isoelectric point values. Our analysis of electrostatic interactions covered three out of the four conceivable cases: when proteins and nanoparticles are charged equally (with the sign “−”) and oppositely (with the sign “+” and “−” or “−” and “+”). We did not consider the variant with the sign “+” and “+”, since we found only one case of interaction of positively charged rod-like gold nanoparticles with trypsin at pH 7.0, at which the protein is positively charged [184].

**Figure 7:**
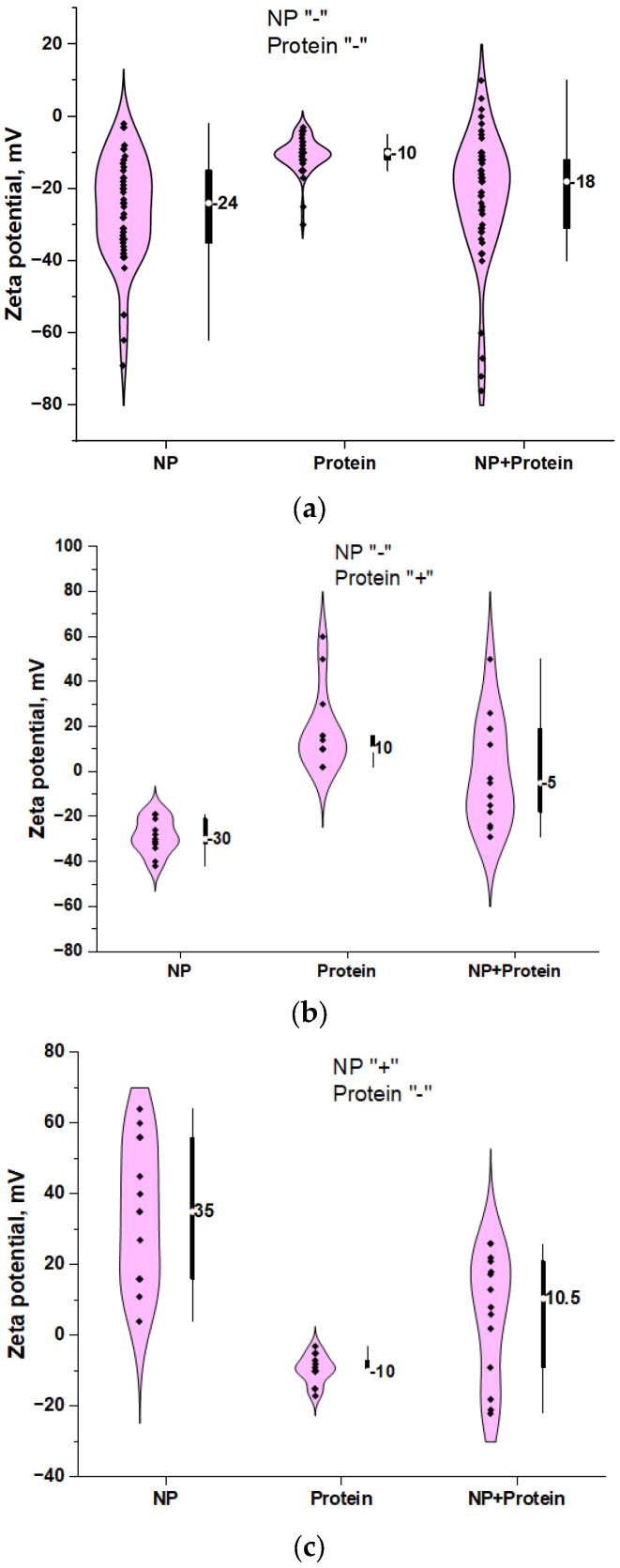
Distributions of ξ-potential values in colloidal solutions of nanoparticles, proteins, and their mixtures in cases where (**a**) nanoparticles are negatively charged, protein molecules are positively charged; (**b**) nanoparticles are negatively charged, protein molecules are negatively charged; (**c**) nanoparticles are positively charged, protein molecules are negatively charged. The graphs show median values of ξ-potentials.

The overwhelming majority of studies (~67%) study the interaction of negatively charged proteins with negatively charged nanoparticles (Figure 7a). Researchers conduct experimental studies at pH close to blood or physiological solution, ~7.4. The isoelectric point of most proteins is at a pH below 7.4, and they carry a negative charge at this pH value. The net charge of the colloidal system typically reaches an intermediate value between the intrinsic charges of the proteins and nanoparticles. Furthermore, the electrokinetic potential profiles exhibit remarkable similarity between bare nanoparticles and those with protein, maintaining comparable distribution shapes and maximum values. It should be noted that the profile of the electrokinetic potential of nanoparticles with protein corona has no similarity with the profile of individual negatively charged nanoparticles or positively charged protein (Figure 7b). At the same time, the level of the electrokinetic potential of nanoparticles with protein corona is much higher than that of positively charged protein molecules. This is likely due to the addition of charge from several protein molecules on the surface of a large nanoparticle. The reaction of a negatively charged nanoparticle with a positively charged protein can produce a product with a significant degree of charge variability. A positive charge on nanoparticles is often achieved by functionalizing the nanoparticles with a CTAB molecule. The number of studies in which positively charged nanoparticles interact with negatively charged protein is about ~17% of the total (Figure 7c). When positively charged nanoparticles interact with negatively charged protein, particles that carry a positive charge in the colloidal solution are often formed (in ~75% of such cases).

## 7. Cytotoxicity of Nanoparticle–Protein Complexes

Nanoparticles must be non-toxic for successful use of in biological applications [247]. The cytotoxicity of nanoparticles is influenced by a large number of factors, especially the physicochemical properties of the surface [248]. Protein adsorption to nanoparticles is crucial for understanding how the cell interacts with the nanoparticle and how the protein corona can regulate cytotoxicity [249]. Gold nanoparticles are generally known to be non-cytotoxic, and this applies only to unliganded nanoparticles [250]. The cytotoxicity of iron oxide nanoparticles is dose-dependent [251]. The cytotoxic effect is the main limitation of the applicability of nanoparticles. Therefore, only a small number of inorganic commercial nanoparticles are commercially available for medical use. Commercially available iron oxide nanoparticles “Nanotherm” are used for local hyperthermia of glioma [252] or “Feraheme” as a contrast agent for MRI [253]. Gold nanoparticle-based drugs such as “AuroLase” and “Aurimune” have received FDA approval for use in medical diagnosis and treatment [254]. Combined gold and silicon nanoparticles (“AuroLase”) are proposed for localized elimination of prostate cancer [255]. The gold nanoparticle-based drug “Aurimune” is undergoing clinical trials for use as antitumor therapy in patients with advanced pancreatic, breast, colon, melanoma, sarcoma, and lung cancer [256].

In general, gold nanoparticles have minimal cytotoxicity. In [225], the absence of cytotoxicity in LLC-Luc cells was demonstrated for gold nanoparticles coated with sodium citrate and passivated with BSA in vitro. Stable HSA corona can reduce the cytotoxicity of silver nanoparticles by suppressing potentially destructive effects on the cell membrane and internalization into RAW 264.7 cells [99]. In another case, the formation of an AGP protein corona on silver nanoparticles preserved the protein’s native conformation, significantly reducing nanoparticle cytotoxicity [58]. Transferrin adsorbed on the surface of titanium dioxide nanoparticles reduces the cytotoxic effect by decreasing the production of intracellular ROS [240]. The cytotoxic effect may also depend on the concentration of the reagent. At low concentrations (1, 10, 50 µg/mL) nickel oxide nanoparticles did not cause significant cytotoxicity of lymphocytes; higher concentrations of nickel oxide nanoparticles (100 µg/mL) caused a significant increase in cell death and the release of lactate dehydrogenase into the intercellular space [236]. Cytotoxicity of iron oxide nanoparticles on fibroblast cells was observed with an increase in the concentration of nanoparticles to 10^13^ particles/mL [161]. It is worth noting that an increase in the size of protein aggregates with nanoparticles was observed at the same concentration.

Collectively, the evidence indicates a general trend of reduced nanoparticle cytotoxicity after protein binding.

## 8. Conclusions

Currently, there is growing interest in the use of inorganic nanoparticles in biomedical research. Numerous studies show that proteins play an important role in the formation of surface coatings on nanoparticles when they enter a biological environment. Another frequently used strategy for biomedical research and applications is the preliminary coating of nanoparticles with proteins to increase the bioavailability of particles. This review discusses the interaction of inorganic nanoparticles of various natures with different protein molecules. It is shown that gold, silver, and iron oxide nanoparticles are used in most studies. The popularity of using gold and silver nanoparticles is explained by their unique optical properties and the abundance of innovative products based on these nanoparticles. Magnetic iron oxide nanoparticles are actively used in biomedicine due to their magnetic properties. The formation of the protein corona is critically determined by the nanoparticle’s chemical identity. Proteins and nanoparticles can reciprocally modify each other’s physicochemical properties, giving rise to novel biological functions in the system.

The size of the nanoparticle is also important in the interaction of the nanoparticle with the protein. The most effective size for nanoparticles is comparable to the size of a protein molecule. Nanoparticles smaller than the size of the protein molecule are apparently ineffective and more often form large aggregates. In any case, when the size of the nanoparticles is smaller than the size of the protein molecules, the use of the term “protein corona” is inappropriate. For reliable determination of complex nanoparticle–protein size, we recommend complementary use of both TEM and DLS methodologies, since they complement each other.

The surface charge of the nanoparticles plays an important role in protein binding. Such environmental parameters as pH, ionic strength, and temperature play an important role in the interaction of nanoparticles with protein molecules. To maximize nanoparticle–protein interaction and achieve the greatest protein corona thickness, experiments must be conducted at the protein’s isoelectric point. However, the strongest interaction of nanoparticles with a protein can be achieved by using them with opposite charges. The concentration of protein, nanoparticles, their ratio in the colloid, and incubation time are also important.

The interaction between nanoparticles and proteins can induce changes in protein structure. The same nanoparticle can affect the structure of one protein and not affect the structure of another. It can be argued that the formation of a covalent bond between a nanoparticle and a protein molecule significantly increases the chances of changing the structure of the latter. Spherical particles less often affect the structure of protein compared to rod-shaped nanoparticles or any other shape with sharp ends. There is a correlation between the effect of changing the structure of protein when interacting with nanoparticles and the negative impact of such nanoparticles on biological systems. There is a tendency to use pre-denatured protein for the functionalization of nanoparticles to reduce the negative effects when entering the biological environment. The relationships between corona thickness, particle size, concentration, and colloidal electrokinetics collectively offer a multidimensional understanding of nanoparticle–protein interplay. The results presented in the review show the possibility of adapting physiological reactions to nanomaterials by engineering the chemical composition of the surface of nanoparticles, size, and charge in accordance with a specific application.

## Figures and Tables

**Table 1:** Characteristics of interaction of proteins with inorganic nanoparticles: protein concentration, colloid pH, buffer, nanoparticle material, shape, size, and zeta potential of the nanoparticle, size and zeta potential of the protein–nanoparticle system, ligands on the surface of the nanoparticles, and constants specified in the work. In the column with zeta potentials, negative values are in brackets. In the column with sizes, the method (TEM, DLS, or SEM) used to measure the size is indicated in brackets.

Protein		Medium		Nanoparticle					Nanoparticle + Protein			Conclusions	Ref.
Type	C_protein_	pH	Buffer	Material	Ligands NP	Shape	Size, nm	ζ, mV	Size, nm	ζ, mV	K		
BSA	8 nM	6.5	SC	Ag	SC	Spherical	15 25 42 (DLS)	−28 −38 −39	16 25 44 (DLS)	−26 −38 −34	K_a_ = 1.7 × 10^7^ M^−1^ K_a_ = 2.1 × 10^7^ M^−1^ K_a_ = 2.2 × 10^7^ M^−1^	A slight increase in the binding constant was observed with an increase in the size of the nanoparticles.	[60]
BSA	2.0 μM	7.4	PBS	Ag	-	Spherical	141 (DLS)	−19	-	-	K_a_ = 6.77 × 10^9^ M^−1^	Stable complexes are formed when BSA interacts with Ag nanoparticles	[208]
BSA	0.06–5 μM	7.4	PBS	Ag	-	Spherical	73 (DLS)	-	-	-	K_a_ = 25.5 × 10^4^ M^−1^	After interaction, a decrease in the content of α-helices was observed; the process was spontaneous (*ΔG* < 0)	[179]
BSA	2 µM–10 mM	7.4	PBS	Ag	SC CTAC SC	Spherical Rods Triangles	85 87 71 (DLS)	-	89 91 64 (DLS)	-	K_d_ = 5.5 × 10^−11^ M^−1^ K_d_ = 1.55 × 10^−10^ M^−1^ K_d_ = 9.57 × 10^−5^ M^−1^	The size and shape of nanoparticles significantly affect the interaction with BSA. In the presence of BSA, nanotriangles gradually evolved into nanodisks.	[180]
BSA	10 μM	7.4	PBS	Ag	PVP Tween 20 CTAB PLL AOT SC	Spherical Triangles Cubic	7–70 (TEM)	-	-	-	K_a_ = 0.07–6 × 10^4^ M^−1^	The observed conformational changes in BSA did not correlate with the Ka value. The interaction of the protein with nanoparticles depends on the interaction of the protein with the functional groups of nanoparticles.	[57]
BSA IgG LYZ	5 μM	7.4	NaCl	Ag	PEI	Spherical	32 (DLS)	+13	55 - -	-	K_a_ = 3.4 × 10^6^ M^−1^ - -	BSA adsorption onto silver nanoparticles functionalized with PEI was observed. The adsorption of IgG and lysozyme did not occur.	[59]
CAT SOD	2 µM 5 µM	7.4	HEPES	Ag	SC	Spherical	20 (TEM) 24 (DLS)	-	33–43 29 (DLS)	-	-	The interaction of CAT with nanoparticles led to changes in the secondary structure of the protein with a loss of enzymatic activity, unlike SOD.	[157]
FBS	-	8.3	DMEM	Ag	SC	Spherical	42 (DLS)	−34	50 (DLS)	−15	K_a_ = 10^5^ M^−1^	The composition and thickness of the protein corona depend on the concentration and incubation time. Hydrophobic interactions are the determining factors of the interaction	[223]
FBS	-	7.4	DMEM	Ag	SC EG_6_OH	Spherical	22 (DLS)	−40	85 (DLS)	−29	-	The composition of serum proteins adsorbed on nanoparticles strongly depends on the density of the EG_6_OH ligand. Functional groups affected toxicity and cellular uptake.	[117]
Hb	1 µg/mL	6.8	Water	Ag	-	Spherical	33 (DLS)	-	100 (DLS)	-	-	Interaction between a protein and nanoparticles leads to an increase in the content of β-layers and a decrease in the number of α-helices	[181]
HSA	0.15 µM	7.3	PBS	Ag	SC	Spherical	20 40 80 (TEM)	−38.8 −32.9 −37.2	24 51 90 (TEM)	−13 −12 −12	K_cat_ = 0.188 s^−1^ K_cat_ = 0.207 s^−1^ K_cat_ = 0.186 s^−1^	The most significant changes in the secondary structure of the protein were observed when HSA interacted with smaller particles.	[99]
HSA	3 μM	7.4	Tris-HCl	Ag	PPh	Spherical	10–20 (DLS)	−36	23–30	−27– (−20)	K_q_ = 2 × 10^10^ M^−1^ × s^−1^	HSA binds to nanoparticles primarily through hydrophobic association; electrostatic interaction is not the main driving force	[209]
AGP	48 μM	7.4	PBS	Ag	SC	Spherical	10 (TEM) 21 (DLS)	−69	25	−76	K_a_ = 10^9^ M^−1^	The formation of a protein corona was accompanied by the preservation of the native protein structure and led to a significant decrease in the cytotoxicity of nanoparticles	[58]
IgG	5 µM–0.5 M	7.0	PBS	Ag	-	Spherical	100–262 (DLS)	-	174–253 (DLS)	−35–(−7)	-	The aggregation and interaction of nanoparticles with protein is influenced by the ionic strength and protein concentration	[142]
LECT2	0–100 ng/mL	7.4	PBS	Ag@ MNP	Apt	Spherical	216 (DLS)	−17	-	−21	-	Compared with the traditional method of random protein immobilization, orientation using an aptamer on Ag@MNP nanoparticles increases the efficiency of binding to the LECT2 protein.	[106]
HSA	25 μM	-	Water	Ag Au Pt	-	Spherical	9 10 10 (DLS)	-	18 21 30 (DLS)	-	-	HSA is considered a biocompatible coating for metal nanoparticles in antibacterial therapy	[61]
HSA	0.1 mg/mL	7.5	PBS	Al_2_O_3_	-	Spherical	118 (DLS)	−31	-	-	K_SV_ = 1.73–5.92 M^−1^	Al_2_O_3_ nanoparticles induce partial unfolding of HSA molecules near aromatic residues, but do not cause significant changes in the secondary structure of HSA even at high concentrations.	[191]
anti-HRP ab	0.03 g/L	6–8.5	PBS, NaOH	Au	SC	Spherical	60 (DLS)	−22	87–200 (DLS)	-	-	The surface charge of the adsorbed protein can be used to control the aggregation of nanoparticles.	[30]
APOA1	5.0 μg/mL	7.4	TE	Au	SC	Spherical Stars	44 58 (DLS)	−39 −35	108 146 (DLS)	−15 −12	-	Precoating of gold nanoparticles with APOA1 protein reduces the expression of ZO-1 in endothelial cells and increases the ability to overcome biological barriers.	[34]
α-Syn	200 μM	7.3	PBS	Au	Nar	Spherical	24 (DLS)	−22	50–100 (DLS)	-	K_a_ = 5.02 × 10^6^ M^−1^	Nanoparticles functionalized with naringenin effectively slow down the aggregation and formation of α-synuclein fibrils.	[197]
BHb EMb	10^−6^ M	7.4	PBS	Au	-	Rods	60 (DLS)	+35	-	18 13	K_a_ = 5.93 × 10^8^ M^−1^	The interaction of proteins with gold nanoparticles causes changes in the secondary structure.	[188]
BLG	50 μM	7.2	PBS	Au	-	Spherical	40 (DLS)	-	52 (DLS)	-	K_a_= 29 × 10^5^ M^−1^	Temperature plays a key role in protein adsorption on the surface of nanoparticles. As the temperature increases, the binding ability decreases, and the amount of adsorbed protein decreases too.	[143]
BSA	15 μM	7.4	PBS	Au	-	Spherical	24 (DLS)	−34	33 (DLS)	−16	-	Exposure to a constant electric field leads to an increase in the thickness of the protein corona in the solution.	[224]
BSA	1 mg/mL	7.4	PBS	Au	PEG	Triangles	90 (DLS)	−27	93 (DLS)	−18	K_sv_ = 3 × 10^8^ M^−1^	The gold nanotriangles retained photothermal properties after interaction with BSA. A decrease in the content of α-helices in the secondary structure was observed	[55]
BSA	0.5–15 μM	6.0	PBS	Au	-	Spherical	30 (DLS)	-	39 (DLS)	−11	-	The protein corona on gold nanoparticles affects the intracellular distribution and retention of particles.	[225]
BSA	1 mg/mL	7.4	PBS	Au	-	Multibranched	125 (DLS)	−24	146 (DLS)	−72	-	The interaction of nanoparticles with the BSA reduces the tendency of nanoparticles to aggregate.	[31]
BSA	0–2 μM	7.4	PBS	Au	CTAB PEG-COOH	Rods	39 × 9.5 (TEM)	56 −17	57 43 (DLS)	22 −22	-	PEG functionalization reduces the adsorption of proteins on the surface of nanoparticles in comparison with CTAB-coated nanoparticles. However, a protein corona still forms on PEG AuNPs, which persists even at high protein concentrations.	[102]
FBG	0–0.4 μM				CTAB PEG-COOH			56 −17	59 43 (DLS)	21 −22			
TM	10–200 nM	-	NaCl	Au	Apt	Spherical	13 (TEM)	-	-	-	-	A spectrophotometric technique has been developed to detect tropomyosin protein at nanomolar concentrations using aptamer-functionalized AuNPs	[226]
Hb	0–100 nM	7.4	PBS	Au	ALA SC	Spherical	25 (DLS)	−28 −21	- 50–80 (DLS)	−24 −18	-	There is a significant loss of the α-helix structure after interaction with citrate nanoparticles.	[107]
HSA	5 μg/L–0.50 g/L	7.4	CBB	Au	-	Spherical	69 (DLS)	-	70 (DLS)	-	-	The colloidal stability of nanoparticles in the presence of a protein is influenced by the concentration of HSA, the ionic strength, and the valence of the cation in the salt solution. The presence of Ca^2+^ promotes additional adsorption of HSA on nanoparticles, which leads to particle aggregation.	[56]
HSA	10/100/200 μM	7.0	Water	Au	CTAB	Rods	35 × 12 (TEM)	-	-	-	-	Changes in the tryptophan microenvironment of the protein in comparison with the native protein are higher at a lower protein concentration.	[184]
HSA	7.5 mM	7.4	PBS	Au	SC PEG-OMe PEG-COOH PEG-NH_2_ Glycan	Spherical	19 45 47 47 35 (DLS)	−14 −2 −9 4 −3	70 55 60 67 79 (DLS)	−15 −5 −12 6 −6	-	Electrostatic interactions and hydrogen bonds play an important role in binding nanoparticles to proteins. Neutral PEG-OMe gold nanoparticles do not cause structural changes, but positively charged PEG-NH_2_ nanoparticles cause conformational changes in HSA at any pH.	[15]
HSA	0–50 μM	7.4	Tris-HCl	Au	PPh	Spherical	17 (TEM)	−20	30 (TEM)	−10	K_b_ = 10^4^ M^−1^	Nanoparticles can have an inhibitory effect on the formation of amyloid fibrils	[227]
HSA	6 μM	7.3	Tris-HCl	Au	CTAB	Stars Rods Flowers	20 (TEM) 28 (DLS) 40 (TEM) 68 (DLS) 42 (TEM) 79 (DLS)	+60 +64 +11	295 (DLS) 220 (DLS) 164 (DLS)	+26 +26 −9	K_a_ = 1.7 × 10^5^ M^−1^ K_a_ = 5.24 × 10^5^ M^−1^ K_a_ = 1.73 × 10^6^ M^−1^	Nanoflowers have a higher ability to interact with HSA compared to other nanoparticles.	[187]
HSA cyt C	0.25 μM 0.9 μM	10.5	CBB	Au	-	Spherical	13 (DLS)	−55	21 18	−21 −60	-	Both proteins have a high affinity for the surface of gold nanoparticles due to free external thiols.	[182]
HSA Histone	0.25–10 μM 0.01–10 μM	7.4	PBS	Au	Phe Lipids	Spherical	30 (DLS)	−42	38 40 (DLS)	−31 50	K_a_ = 4.22 × 10^6^ M^−1^ K_a_ = 1.18 × 10^4^ M^−1^	The functionalization of the nanoparticle surface by fats provides increased resistance to protein adsorption.	[120]
HSA TF	1 mg/mL	7.4	PBS	Au	DHLA	Spherical	1.7 (TEM)	−20	34 (DLS) -	−40– (−15) −35 (−10)	-	Nanoparticles have little effect on the secondary structure of HSA and no effect on the secondary structure of transferrin.	[101]
FBG TRP	10^−11^–10^−4^ M	7.0	PBS	Au	SC	Spherical	35 (TEM)	−31.7	-	−25 −10.6	K_d_ = 1.58 × 10^8^ M^−1^ K_d_ = 6.45 × 10^7^ M^−1^	Nanorods and nanostars can induce large changes in the secondary structures of proteins, unlike spherical nanoparticles.	[183]
FBG TRP					CTAC	Rods	48 × 14 (TEM)	+44.8		+17.3 +36.5	-		
IgG	1 μM	7.4	PBS	Au	SC	Spherical	60 (TEM) 69 (DLS)	−21	94 (DLS)	−11	-	The biological effect of the protein corona can be manipulated by changing the structure of proteins.	[32]
Insulin	10 μM	7.4	PBS	Au	Pro HydroxyPro	Spherical	10 (DLS)	−72 −45	-	-	K_a_ = 1.303 × 10^4^ M^−1^ K_a_ = 0.9904 × 10^3^ M^−1^	Osmolite-functionalized nanoparticles contribute to the preservation of the native protein structure under aggregation conditions.	[126]
Insulin	20 μM	3.0, 7.4	Gly-HCl PBS	Au	PEG_200_ PEG_1500_ PEG_6000_ PEG_10000_	Spherical	56 59 51 50 (DLS)	−25 −20 −10 −5	65 78 141 105	-	-	Longer fibrils were formed when the protein interacted with nanoparticles that were functionalized by PEG with a higher molecular weight.	[33]
S-protein SARS-CoV-2	5 ng/µL	7.5	PBS	Au	BSPP	Spherical	52 (TEM) 121 (DLS)	-	93 (TEM) 173 (DLS)	-	-	The conjugate of protein and AuNPs causes a strong reaction of antigen-specific IgG.	[212]
TF	2 mg/mL	7.4	PBS	Au	SC PEG TPN Cys GME	Spherical	5 23 6 21 7 (DLS)	−34 −24 −24 −9 0.4	17 24 14 39 6	−26 −24 −32 −18 −2	-	Functionalization of nanoparticles with glutathione monoethyl ether resulted in resistance to protein adsorption and aggregation, as well as low cytotoxicity compared to other functional groups.	[119]
BHb Lys	0.08–2.40 × 10^−8^ M	-	Water	Au/Ag	SC	Spherical	24 (DLS)	−31	32 75 (DLS)	−10 −5	K_sv_ = 5.7 × 10^9^ M^−1^ K_sv_ = 5.35 × 10^9^ M^−1^	Nanoparticles influenced the structure of the BHb and Lys proteins	[189]
SP-B analog	4 µg/mL	5.2–6.4	TFE/ PBS	Au	-	Spherical	5 10 20 (DLS)	−17 −13 −15	139 85 131	−11 5 10	-	The content of the α-helical structure in the protein decreased when interacting with nanoparticles, and the efficiency of the process depends on the size.	[100]
				Ag			10 (DLS)	−9	126	+2			
BSA	4 µM	-	Water	C_60_	T80	Spherical	243 (TEM)	−13	357	−14	-	The C_60_ nanocomplex affects the secondary structure of the protein. The adsorption of BSA proceeded continuously, while FBG was partially desorbed after 4 h	[228]
FBG	0.6 µM								412	−22			
Hb	4 µM								540	+9			
LYZ	50 µg/mL	6.2	PBS	C_60_	NaOH	Spherical	0.67 nm (DLS)	-	-	-	-	Fullerene functionalized by a hydrophilic group suppresses the enzymatic activity of lysozyme	[229]
BSA	2 µM	7.0	PBS	C	-	Spherical	360 (DLS)	-	343	-	K_q_ = 0.98 × 10^7^ L mol^−1^ s^−1^	BSA coating decreases carbon nanoparticle cytotoxicity against BEAS-2B cells and is accompanied by a slight reduction in the protein’s α-helical content	[230]
HSA	0.15 mM	7.2–7.4	MES TRIS	Cr_2_O_3_	-	Spherical	100 (TEM)	−18.29	-	−10.66	-	BSA binds to nanoparticles in a preferred orientation due to electrostatic interaction	[192]
α-La	2.5 μM	-	Water	Fe_3_O_4_	Cellulose	Spherical	14 (DLS)	−23	-	−32	K_a_ = 9.68 × 10^11^ M^−1^	There are no conformational changes in the protein during interaction with nanoparticles. There is a strong interaction with protein during the functionalization of magnetic particles by cellulose in comparison with magnetic particles.	[231]
Anti-BSA	6 nM	7.4	PBS	Fe_3_O_4_	BSA	Spherical	89 (DLS)	-	100 (DLS)	-	Ka = 1.3 × 10^5^ M^−1^ × s^−1^	Iron oxide nanoparticles can be used to extract the target protein. The kinetics of binding were measured in solution without elution or reimmobilization.	[70]
BGL	5 mg/mL	4–9	PBS	Fe_3_O_4_	SA	Spherical	10–40 (DLS)	-	40–50 (DLS)	-	K_cat_ = 20.86 s^−1^	The enzyme was more stable when interacting with iron oxide nanoparticles at different pH and temperature than the enzyme in the absence of nanoparticles under the same conditions.	[71]
BSA	1 mg/mL	6.0	PBS	Fe_3_O_4_	GA	Spherical	38 (DLS)	−24.5	98 (DLS)	−16.7	-	Nanoparticles in the presence of BSA were heated more efficiently than nanoparticles without BSA and showed significant internalization in cancer cells.	[69]
BSA	0–50 µM	7.4	PBS	Fe_3_O_4_	SC	Spherical	18 (TEM) 30 (DLS)	−28.2	37 (DLS)	−18.4	K_a_ = 4.6 × 10^5^ M^−1^	Denaturation of BSA in interaction with nanoparticles leads to a transition from the formation of a monolayer on nanoparticles to the formation of stable complexes.	[205]
FBS	-	7.4	PBS	Fe_3_O_4_	PVA	Spherical	8 (TEM)	+2	150–276 (DLS)	−4	-	Proteins responsible for the long-term circulation of nanoparticles were found: osteopontin, lipoprotein lipase, coagulation factor VII, matrix protein GLA, secreted phosphoprotein 24, alpha-2H glycoprotein, and apolipoprotein C-I.	[124]
HEWL	0.01–5 mg/mL	4–11.8	Water NaOH	Fe_3_O_4_	SC	Spherical	3 (DLS)	−32	246–462 (DLS)	−25–(+10)	-	Nanoparticles are involved in the formation of large aggregates, the size of which depends on the concentration of protein and nanoparticles. The addition of HEWL to clusters of nanoparticles leads to the disintegration of clusters into individual nanoparticles.	[161]
HSA	2 mg/mL 8 mg/mL	6.0–7.5	PBS	Fe_3_O_4_	-	Spherical	50 (DLS)	-	90 (DLS)	-	K_a_ = (1.3–5.8) × 10^4^ M^−1^	The interaction of HSA with iron oxide nanoparticles depends on the pH and ionic strength in the solution.	[138]
HSA	0.29–0.91 g/L	7.4	HEPES	Fe_3_O_4_	L-PEOXA C-PEOXA	Spherical	52 21 (DLS)	-	60 10–50 (DLS)	-	-	Cyclic PEOXA quantitatively prevents protein adsorption on nanoparticles. However, the dense shell of linear PEOXA cannot prevent a weak but significant interaction with HSA.	[118]
HSA TF	0.2 mg/mL	7.4	PBS	Fe_3_O_4_	Bilayer of OA and 18 LPC	Spherical	16 (DLS)	−8	22 20–40 (DLS)	−17 −17	K_a_ = 6.2 × 10^8^ M^−1^ -	The protein structure did not change significantly after interaction with nanoparticles. The conjugate of HSA with nanoparticles did not affect the properties of the lipid bilayer.	[62]
IgG	0.6 mg/mL	3.0–9.0	AC CBB PBS	Fe_3_O_4_	κ-carrageenan	Spherical	70 (TEM)	−25	-	-	-	Magnetic nanoparticles may be a promising material for IgG isolation and purification.	[232]
IgG	0.12–4.8 mg/mL	6.6	Tris-HCl	Fe_3_O_4_	SC	Spherical	50 (TEM)	-	-	-	-	Structural and conformational changes in the protein were observed when IgG interacted with nanoparticles.	[74]
OVA	2.93 mg/mL	7.4	PBS	Fe_3_O_4_	Methyl-modified	Spherical	10 (DLS)	−38	251 (DLS)	−26	-	Iron oxide nanoparticles were used for magnetic separation of ovalbumin from the medium.	[233]
TRP	0.75 mg/mL	4–11	SC PBS CBB	Fe_3_O_4_	PVA	Spherical	244 (DLS)	−22	-	-	-	Immobilized trypsin showed efficient proteolytic activity within a shorter period (15 min) compared to free trypsin (24 h)	[234]
CRP	0.1–40 mg/L	7.4	Tris-HCl	Fe_3_O_4_@ SiO_2_/ COOH	EDTA/ TMS	Spherical	53 (TEM) 750 (DLS)	−65	920 (DLS)	−65	-	The effect of protein concentration on changes in the hydrodynamic characteristics of nanoparticles is shown. Such nanoparticles can be used for magnetic separation of proteins from solution.	[235]
Hb	3 μM	7.4	Tris-HCl	NiO	-	Spherical	20–50 (TEM) 175 (DLS)	−29	-	-	K_sv_ = 3.24 × 10^4^ M^−1^	It has been shown that nanoparticles interact with hydrophilic amino acid residues in hemoglobin.	[236]
BSA HEWL	1 mg/mL	4.0–9.0	AC BBS	TiO_2_	SC	Spherical, cubical	21 (TEM)	−11.6	-	-	K_d_ = 130–500 M^−1^ K_d_ = 750–2000 M^−1^	The binding affinity of TiO_2_ nanoparticles with lysozyme is higher than with BSA.	[136]
LYZ	0.1–0.2 mg/mL	6.2	PBS	Se	SC	Spherical	35 (SEM) 71 (DLS)	−30.2	84 (DLS)	3	-	The synergistic antibacterial activity of SeNPs and lysozyme against *E. coli* and *S. aureus* has been shown.	[237]
α-Syn	100 µM	7.4	HEPES, NaCl	SiO_2_	-	Spherical	20 (TEM)	-	-	-	k_a_ = 0.083 ± 0.011 h^−1^	The presence of nanoparticles accelerates the amyloid fibrillation of the protein. Amyloid fibrils formed after interaction with nanoparticles were more toxic in comparison with those obtained without nanoparticles.	[194]
BHb	2–10 μM	7.4	PBS	SiO_2_	-	Spherical	95 84 (DLS)	18 40	165–190 320–370 (DLS)	6 24	-	The nanoparticles induced conformational changes in the protein around Tyr residues and heme degradation.	[80]
BSA	10^−7^–10^−3^ M	7.4	PBS	SiO_2_	-	Spherical	9 (TEM)	-	-	-	-	Significant adsorption of BSA on nanoparticles was observed.	[238]
BSA LYZ BGG	27.2 g/L 19.1 g/L 0.119 g/L	7.4	PBS	SiO_2_	-	Spherical	23 (TEM) 31 (DLS)	−39	250 5000 3000	-	-	The authors proposed an approach to differentiate between different types of aggregates.	[19]
Mb	0.1–5 mg/mL	4.0–10.0	Water	SiO_2_	-	Spherical	30 (DLS)	−45	-	-	-	Changing the ionic strength of the solution allows you to control the adsorption–desorption of protein on the surface of the nanoparticle.	[140]
Mb Hb HbA1c HbAm	0.01–2 g/L	7.0	PBS	SiO_2_	-	Spherical	33.6	−17	-	-	K_a_ = 1.7 × 10^5^ K_a_ = 1.9 × 10^6^ K_a_ = 1.1 × 10^6^ K_a_ = 5.7 × 10^6^	Large proteins interacted with nanoparticles with greater affinity than small proteins.	[206]
oxyHb	1 mM	7.0	PBS	SiO_2_	-	Spherical	26 (DLS)	−42	30 (DLS)	-	K_a_ = 2.1 × 10^5^ M^−1^	Nanoparticles affect the secondary structure of weakly bound proteins	[29]
BSA	0.51 mM	2–8	Water	SiO_2_ TiO_2_ ZnO	-	Spherical	155 493 520	−32 −28 −31	162 475 147	−38 −31 −31	K_a_ = 1.1 × 10^5^ M^−1^ K_a_ = 1.3 × 10^6^ M^−1^ K_a_ = 1.8 × 10^5^ M^−1^	The affinity of the interaction of BSA with nanoparticles decreases in the line: ZnO > SiO_2_ > TiO_2_. The interaction between the particles and the protein is spontaneous (*ΔG* < 0). Minor changes in the secondary structure of the protein were observed during adsorption on nanoparticles.	[82]
WP	100 μg/mL	2–11	Water	TiO_2_	-	Spherical	20–40 (TEM)	−12	-	−18	-	The formation of a protein corona on the surface of nanoparticles increases their antioxidant activity, which can reduce the negative biological effects of nanoparticles.	[135]
ALD CAT LDH QOR	1 mg/mL	7.5	PBS	Al_2_O_3_ ZnO Fe_3_O_4_ CuO NiO	-	Spherical	40 35–45 35–45 70 40 (DLS)	-	-	-	-	The effect of copper nanoparticles had a significant effect on the conformation, stability, and activity of metabolic enzymes in comparison with other nanoparticles.	[84]
BSA	1 mg/mL	2–12	Water	CeO_2_ TiO_2_ ZnO	-	Spherical	58 68 78 (DLS)	−30–(+43) −26–(+33) −69–(+13)	-	-	-	The content of the α-helical structure decreases, and the content of the disordered structure of BSA increases with the addition of nanoparticles.	[239]
BSA	100–1000 μM	-	Water	TiO_2_	-	Cubic	130–260 (DLS)	−38	500 (DLS)	−30	K_a_ = 8.76 × 10^2^ M^−1^	The values of hydrodynamic diameters and charges in colloids are important variables for analyzing the effect of the ionic force on the colloidal properties of nanoparticles.	[207]
Pepsin	3.2 mg/mL	1.2	HCl NaCl	TiO_2_	-	Spherical	163 (DLS)	+27	208 (DLS)	+2	-	Nanoparticles have no noticeable effect on the secondary structure of pepsin. However, the interaction of pepsin with nanoparticles led to a decrease in enzyme activity.	[35]
Luc	3 µM	7.4	Tris–AC	TiO_2_	-	Spherical	24 (DLS)	−11	50 (DLS)	−4	-	The interaction of luciferase with nanoparticles leads to a change in the secondary structure of the protein and promotes invalid folding of luciferase.	[193]
TF	2.5 mg/mL	7.4	PBS	TiO_2_	-	Spherical	30–50 (TEM)	-	50–70 (TEM)	-	-	Modification of the surface of nanoparticles with protein increased the stability of nanoparticles and their penetration into cells.	[240]
BSA Collagen Zein LYZ	0.02 mg/mL	3.5–10.4	Water NaOH	TiO_2_	-	Spherical	-	-	80, 1500 10–60 10–60 5–30 (DLS)	-	-	The greatest absorption by SW1417 cells was observed for titanium nanoparticles functionalized with BSA.	[85]
BLG	-	7.4	PBS	TiO_2_	-	Spherical	3200	-	400	-	-	The interaction of nanoparticles with β-lactoglobulin affects the secondary structure of the protein. There are no changes in the protein structure when nanoparticles interact with gelatin. It has been established that β-lactoglobulin promotes more efficient penetration of nanoparticles into cells than gelatin.	[86]
BSA	2–16 mg/mL	7.0	PBS	TiO_2_	-	Spherical	300 (DLS)		200–250 (DLS)	-	-	The concentration of BSA affected the thickness of the adsorbed layer. Spatial repulsion caused by an increase in the thickness of the adsorption layer of the protein increases the stability of the nanoparticles.	[241]
BSA	100 μg/mL	4–5	Water HCl NaOH	TiO_2_	-	Spherical	600 (DLS)	+41	2500 (DLS)	−18	-	There is an increase in the percentage of β-structures and a decrease in α-helices in the BSA structure when interacting with nanoparticles.	[195]
GN GD SPI Zein	100 mg/mL	7.0 7.0 9.0 7.0	water	TiO_2_	-	Spherical	32 (TEM) 760 (DLS)	−18.7	180 2733 300 200 (DLS)	+22.6 +61.4 −15.6 +18.9	-	There is an increase in the percentage of β-structures in proteins when proteins bind to TiO_2._	[211]
TRP	0.01 g/mL	7.4	PBS	TiO_2_	-	Spherical	60 (TEM) 100 (DLS)	0.7	-	-	K_sv_ = 1.56 × 10^6^ M^−1^	The content of β-sheets in trypsin decreased when interacting with nanoparticles, while the content of random structures increased when the protein binds to TiO_2._	[121]
BSA FBG	2 mg/mL	7.4	PBS	ZnO	-	Spherical	50 (DLS)	-	806 30 (DLS)	-	-	The interaction of nanoparticles with fibrinogen avoids the effect of nanoparticle agglomeration.	[83]
BSA Casein Zein	1 mg/mL	7.4	Water	ZnO	-	Spherical	78 (SEM)	+16	380 600 1600 (DLS)	−22 −21 +8	- K_a_ = 2.1 × 10^7^ M^−1^ K_a_ = 2.2 × 10^7^ M^−1^	Cellular uptake increases with the functionalization of ZnO nanoparticles by BSA protein relative to nanoparticles without protein.	[210]
BSA	10 mM	7.4	PBS	ZnO	Pro	Spherical	702 (DLS)	−12	10 (DLS)	-	K_b_ = 0.3875 M^−1^	Zinc oxide nanoparticles interact to a greater level with hydrophobic protein groups. A mixture of ZnO and proline at high temperature promotes protein fibrillation.	[129]

## Abbreviations

The following abbreviations are used in this manuscript:

ab	Antibody
α-syn	α-synuclein
α-La	α-Lactalbumin
AC	Acetate buffer
AGP	Human α-1-acidglyco-protein
ALA	α-lipoic acid
ALD	Aldolase
AOT	Sodium bis(2-ethylhexyl) sulfosuccinate
APOA1	Apolipoprotein A1
APOH	Apolipoprotein H
Apt	Aptamer
BBS	Borate Buffered Saline
BGG	Bovine γ-globulin
BGL	β-glucosidase
BHb	Bovine hemoglobin
BLG	β-lactoglobulin
BSA	Bovine Serum Albumin
BSPP	Bis(2-sulfonatophenyl)phenylphosphine
CAT	Catalase
CBB	Carbonate-Bicarbonate Buffer
CD	Circular dichroism
GN	Glutenin
CRP	C-reactive protein
CTAB	Cetyltrimethylammonium bromide
CTAC	Cetrimonium chloride
CT	Chymotrypsin
Cys	Cysteine
Cyt C	Cytochrome C
DHLA	Dihydrolipoic Acid
DLS	Dynamic light scattering
DMEM	Dulbecco’s Modified Eagle Medium
EGFP	Green fluorescent protein
EG6OH	(11-mercaptoundecyl)hexa(ethylene glycol)
EMb	Equine skeletal myoglobin
FBG	Fibrinogen
FBS	Fetal Bovine Serum
FTIR	Fourier Transform Infrared Spectroscopy
GA	Glutaric acid
GD	Gliadin
Gly	Glycine
GME	Glutathione monoethyl ester
Hb	Hemoglobin
HbA1c	Riftia pachyptila coelomic hemoglobin
HbAm	Arenicola marina hemoglobin
HEPES	4-(2-Hydroxyethyl)piperazine-1-ethanesulfonic acid
HEWL	Hen egg white lysozyme
HSA	Human Serum Albumin
IgG	Immunoglobulin G
LECT2	Leukocyte cell-derived chemotaxin-2
LDH	Lactate dehydrogenase
18 LPC	1-Palmitoyl-2-lysophosphatidylcholine
LYZ	Lysozyme
Luc	Luciferase
Mb	Myoglobin
MES	2-(N-morpholino)ethanesulfonic acid
MRI	Magnetic resonance imaging
Nar	Naringenin
OA	Oleic acid
oxyHb	Oxyhemoglobin
OVA	Ovalbumin
PBS	Phosphate-Buffered Saline
PEG	Polyethylene Glycol
PEI	Polyethylenimine
C-/L-PEOXA	Linear/cyclic poly(2-ethyl-2-oxazoline)
Phe	Phenylalanine
PLL	Poly-L-lysine
PPh	Polyphenols
Pro	Proline
PVA	Polyvinyl alcohol
PVP	Polyvinylpyrrolidone
QOR	Quinone oxidoreductase
SA	Sodium alginate
SOD	Superoxide dismutase
SP-B	Surfactant Protein B
SPI	Soy Protein Isolate
SPR	Surface plasmon resonance
SC	Sodium Citrate
T80	Tween 80
TE	Solution of Tris and EDTA
TEM	Transmission electron microscope
TF	Transferrin
TFE	Trifluoroethanol
TM	Tropomyosin
Tris-HCl	Tris(hydroxymethyl)aminomethane Hydrochloride
TPN	Tiopronin
TRP	Trypsin
Trp214	Amino acid residue of tryptophan
WP	Whey protein
